# Bipolar disorder integrative staging: incorporating biomarkers into progression across stages (BOARDING-PASS) – rationale and design

**DOI:** 10.3389/fpsyt.2025.1646533

**Published:** 2025-10-31

**Authors:** Nicolaja Girone, Laura Cremaschi, Monica Macellaro, Camilla Gesi, Francesca Martella, Salvatore Saluzzi, Stefano Martinelli, Andrea Caporali, Ilenia Rosa, Clara Cavallotto, Annabella Di Giorgio, Claudio D’Addario, Giovanni Martinotti, Mauro Pettorruso, Mauro Gianni Perrucci, Francesco de Pasquale, Simonetta Gerevini, Emi Bondi, Bernardo Dell’Osso

**Affiliations:** ^1^ Department of Mental Health, L. Sacco Hospital, University of Milan, Milan, Italy; ^2^ Department of Biomedical and Clinical Sciences, L. Sacco Hospital, University of Milan, Milan, Italy; ^3^ Department of Bioscience and Technology for Food, Agriculture and Environment, University of Teramo, Teramo, Italy; ^4^ Department of Mental Health and Addictions, ASST Papa Giovanni XXIII, Bergamo, Italy; ^5^ Faculty of Veterinary Medicine, University of Teramo, Teramo, Italy; ^6^ School of Advanced Studies, Center for Neuroscience, University of Camerino, Camerino, Italy; ^7^ Department of Neuroscience, Imaging and Clinical Sciences, University G. D’Annunzio Chieti-Pescara, Pescara, Italy; ^8^ Neuroradiology Unit, Azienda Socio Sanitaria Territoriale (ASST) Papa Giovanni XIII, Bergamo, Italy; ^9^ “Aldo Ravelli” Center for Neurotechnology and Brain Therapeutics, University of Milan, Milan, Italy; ^10^ Department of Psychiatry and Behavioral Sciences, Bipolar Disorders Clinic, Stanford University, Stanford, CA, United States; ^11^ Neuroscience Research Center, Department of Biomedical and Clinical Sciences, University of Milan, Milan, Italy

**Keywords:** bipolar disorder, neuroprogression, staging models, epigenetic, inflammation, machine learning, biomarkers, neuroimaging

## Abstract

Bipolar disorder (BD) is a lifelong, recurrent condition with growing evidence supporting a neuroprogressive course, entailing the need to adopt staging models to guide stage-specific interventions. Although different approaches have been proposed, their application remains limited and largely based on clinical features. BOARDING-PASS is an Italian government-funded, multicenter, prospective, and observational study aimed at advancing current knowledge of BD progression through the integration of clinical, biological, neuroimaging data, alongside machine learning (ML) methodologies. The study enrolled 97 subjects (age 18–70 years), classified according to the Kupka & Hillegers’ staging model, and recruited from three secondary-level psychiatric services in Italy. The primary outcome is the longitudinal assessment of clinical stage progression over an 18-month period, with evaluations conducted at baseline (T0), T1 (6 months), T2 (12 months), and T3 (18 months after baseline). At each time point, clinical variables will be collected, as well as clinical stages assigned. Additionally, at T0, T2, and T3, peripheral blood and unstimulated saliva samples will be collected to assess epigenetic regulation of gene expression - including DNA methylation, histone modifications, and exosomal miRNAs - with a focus on key biomarkers such as C-reactive protein, proinflammatory cytokines, and BDNF, as well as microbial signatures of major oral bacterial phyla. Structural and resting-state functional MRI scans will also be acquired at the same time points: structural data will be used to compute the structural connectome based on gyrification-based covariance networks, while resting-state data will be used to assess functional connectome alterations via graph theory metrics. Finally, all multimodal data will be integrated within a supervised ML algorithm based on Support Vector Machine, with the goal of developing a refined, data-driven staging model for BD. BOARDING PASS project aligns with the growing need for a standardized, biologically informed staging framework that integrates clinical, inflammatory, epigenetic, and neuroimaging profiles to enhance prognostic accuracy and support tailored therapeutic interventions in BD.

## 1 Introduction and background

Bipolar disorder (BD) is a severe, chronic, and recurrent psychiatric disorder, affecting 1-3% of the global population. It is associated with relevant morbidity and economic burden (1): indeed, WHO’s World Mental Health Surveys ranked BD as the second most disabling psychiatric illness in terms of lost productivity (2). Nonetheless, BD diagnosis and management remain challenging, primarily due to its heterogeneous presentation, the high rate of frequent comorbidities, and the absence of reliable biomarkers of disease progression.

According to the (51), BD prevalence has been stable worldwide and is comparable to that of schizophrenia, underlining its high heritability, with genetic heritability estimated around 70–90% (4). Although genome-wide association studies (GWAS) have identified multiple risk loci, each of them exerts only minor effects, supporting the notion of a multifactorial model in which gene-environment interactions contribute to disease vulnerability (5). In fact, the pathophysiology of BD involves a complex interplay of genetic, neurobiological, and environmental factors that lead to epigenetic, endocrine, and inflammatory dysregulation. These mechanisms drive neuronal alterations that contribute to illness onset and progression (5–7). Within this framework, the concept of neuroprogression has been proposed, positing that BD may follow a progressive trajectory marked by cumulative molecular, neuroanatomical, and functional changes that contribute to clinical worsening (8). Evidence supporting this hypothesis comes from neuroimging studies reporting progressive cortical thinning, hippocampal atrophy, and disruptions in white matter integrity, particularly in patients with multiple episodes and prolonged illness duration (9). On a molecular level, BD progression has been associated with altered levels of Brain-Derived Neurotrophic Factor (BDNF), inflammatory cytokines, and oxidative stress markers, which fluctuate depending on illness stage and symptomatic state (3, 10, 11, 13). From a clinical perspective, this process manifests as increased susceptibility to recurrent affective episodes, reduced likelihood of sustained remission, as well as progressive cognitive and functional impairment (5, 14, 15). However, the notion of neuroprogression in BD remains debated (16, 17). Recent prospective longitudinal studies have questioned the consistency of progressive decline. For example, first-treatment BD cohorts followed for ~10 years show long-term cognitive stability or improvement with trajectories paralleling healthy controls, and no evidence of kindling or cumulative deterioration (18, 19). Taken together, these findings underscore the heterogeneity of evidence and highlight the need for further multimodal longitudinal studies capable to clarify illness-related progression.

In light of these considerations, clinical staging models have been developed to better characterize BD progression and guide stage-specific interventions. The natural course of illness typically includes an asymptomatic at-risk phase, followed by a prodromal stage with non-specific symptoms, a first syndromic mood episode, and subsequent recurrences without full inter-episode recovery (20, 21). Despite theoretical advancements, the clinical applicability of BD staging models remains limited, with only a few studies assessing their real-world feasibility. In 2019, Van der Markt and colleagues applied the Kupka & Hillegers’ model to a sample of BD outpatients, confirming progressive illness trajectories and identifying biphasic mood episodes at onset and male gender as risk factors for faster progression (22). A follow-up study by Van der Markt and colleagues in 1396 BD I patients strengthened the construct validity of staging frameworks but highlighted inconsistencies between classification systems, suggesting the need for refined stratification approaches (23). In the same year, a Spanish group applied a k-means clustering approach integrating clinical, cognitive, and functional measures in a sample of 224 BD patients, 129 of whom followed for three years, showing functional deterioration and increasing treatment complexity with stage progression (24). More recently, Macellaro and colleagues from our research group conducted a ten-year retrospective study of multiple BD staging models. The findings further supported the hypothesis of significant stage progression and highlighted the influence of age at first elevated episode and duration of untreated illness on stage advancement (25). Expanding upon this, Cremaschi and colleagues focused on the Kupka & Hillegers staging model and applied a multistate Markov model to estimate transition probabilities across different BD stages. Notably, a high hazard of transition from stage 2 to 3 was observed, with the probability of remaining in stage 2 decreasing to 14% after three years (26).

Beyond clinical symptomatology, pharmacological treatments has emerged as a critical modulator of disease progression in BD. Current international guidelines, including those from the Canadian Network for Mood and Anxiety Treatments (CANMAT) and the International Society for Bipolar Disorders (ISBD), recommend first-line pharmacological strategies for both acute phases (mania and bipolar depression) and long-term maintenance, typically starting with lithium or valproate, either as monotherapy or in combination with atypical antipsychotics (27, 28). As extensively reviewed by Berk et al. (29), lithium has been shown to reduce inflammation, counteract oxidative stress, and promote neurotrophic activity, especially through effects on BDNF expression and methylation (29, 30). Early intervention through timely diagnosis, comprehensive biopsychosocial treatment, recognition of neuropsychiatric and physical comorbidities, and personalized management is likely to be more effective and to improve long-term outcomes (28, 31). In this regard, there is also consistent evidence that early pharmacological intervention may attenuate illness progression by reducing relapse rates and cognitive decline (32–34). Furthermore, treatment adherence has consistently been identified as a major determinant of illness trajectory, with poor adherence linked to higher relapse rates, increased suicidality, and poorer functional outcomes, particularly in the early stages of BD (35). These findings support the importance of accounting for pharmacological variables and adherence to treatment when interpreting biomarker-based staging outcomes.

Despite ongoing efforts, BD staging may be daunting, primarily due to the lack of reliable biomarkers for illness vulnerability and progression (29, 36, 37). In this perspective, recent advances in biological markers, neuroimaging, and computational models now offer an opportunity to enhance the precision and applicability of clinical staging models, enabling more personalized patient care and tailored treatment.

In this context, the BOARDING PASS study aims to refine clinical staging in BD by integrating traditional frameworks with advanced biological and neuroimaging data. Specifically, this multi-site, longitudinal, observational study adopts Kupka & Hillegers’ staging model (38), enriched with biomarkers such as BDNF, inflammatory, and epigenetic markers, as well as structural and functional neuroimaging data. Moreover, supervised machine learning (ML) algorithms will be trained on the resulting multimodal dataset to support biologically-informed, personalized risk stratification, early detection of the disorder, and the development of precision treatment strategies.

## 2 Specific aims and experimental design

BOARDING PASS study has the following objectives:

- to longitudinally assess BD clinical progression over an 18-month period using the Kupka & Hillegers’ staging model;
- to investigate the role of biological and neuroimaging markers in BD stage transitions (i.e. gene transcription regulation, inflammation, microbiotic, structural and functional neuroimaging measures);
- to implement a predictive ML model based on the integration of clinical, biological, and neuroimaging data, in order to provide an individualized and data-driven prediction of BD stage transitions.

A total of 126 subjects have already been enrolled across three Italian recruiting sites: Unit 1 (ASST Fatebenefratelli-Sacco, Milan; n=44), Unit 2 (ASST Papa Giovanni XXIII, Bergamo; n=41), and Unit 3 (ASL 2 ABRUZZO Lanciano-Vasto-Chieti; n=41). Participants are currently undergoing an 18-month follow-up, with 3 time points: T1 (6 months after baseline), T2 (12 months after baseline), T3 (18 months after baseline). At each time point, the clinical stage will be assigned according to the Kupka & Hillegers’ staging model, and changes in a subset of baseline clinical variables will be assessed to register potential progression in clinical staging.

Additionally, a set of psychometric measures will be administered. To investigate gene transcription regulation, pro-inflammatory cytokines (e.g., TNF-α, IL-6, IL-19), and BDNF profiles, biological samples will be collected at T0 and subsequently at T2 and T3. Biological samples from each recruitment site will be centralized at Unit 4 (University of Teramo) for standardized processing and analysis.

Neuroimaging assessments, including structural (sMRI) and resting-state functional MRI (rs-fMRI), will be conducted at each site at T0, T2, and T3, to evaluate structural connectomes based on the gyrification indices and resting-state functional connectivity. MRI datasets from all sites will be centralized at UO3 for standardized pre-processing and analysis, conforming to ENIGMA Network standards (39).

Finally, ML algorithms will be developed using MATLAB’s Statistics and Machine Learning Toolbox. A range of supervised ML models will be explored to identify the most accurate configuration. Support Vector Machines and Bayesian approaches will be tested to train the models on multimodal datasets comprising clinical, biological and neuroimaging markers.

## 3 Major endpoints

The primary outcome of the BOARDING PASS study is the longitudinal assessment of clinical stage progression in BD at 6, 12, and 18 months, according to the Kupka & Hillegers’ staging model. Recent findings from our research group (25, 26) have supported the feasibility and sensitivity of the Kupka & Hillegers’ model in reporting significant stage transitions over time. Furthermore, the potential association between stage progression and socio-demographic and/or clinical characteristics will be investigated.

Secondary endpoints include the evaluation of biological and neuroimaging characteristics throughout the course of illness, along with their predictive power on illness progression. Specifically:

- Gene Transcription Regulation: previous evidence reported reduced BDNF levels during mood episodes, correlated with hypermethylation of the BDNF promoter region (12, 30). Additionally, higher methylation levels were found in depressive states compared to manic or mixed episodes, with early-onset BD being specifically associated with increased methylation at the BDNF Val66Met polymorphic site (41). Based on this, BDNF levels and/or BDNF gene expression downregulation are expected to correlate with BD stage progression.
- Inflammation: alterations in pro-inflammatory cytokines (e.g., IL-1β, IL-6, TNF-α) may be correlated to clinical progression and neuroplasticity changes (29).
- Microbiota: as recent insights suggest a potential microbiota-host epigenetic axis in neuropsychiatric disorders, further evidence may be added on the possible interplay between oral microbiota modulation and epigenetic regulation of gene expression in different BD stages (40, 42).
- Neuroimaging: consistent data so far indicated structural and functional brain alterations in BD patients and their unaffected relatives (44). Cortical morphological changes will be assessed through gyrification-based structural covariance networks. We hypothesize a progressive reduction in assortativity (a measure of resilience of the connectome) and in transitivity (indicating increased segregation) across BD stages. In terms of functional data, we expect to observe abnormal functional connectivity patterns in large-scale brain networks along BD progression. In particular, the Default Mode Network has consistently shown altered patterns of hyper- and hypo-connectivity with affective, fronto-parietal and attentive systems, disrupting efficient large-scale brain communication. Together, gyrification-based structural connectome and functional connectivity at rest could represent reproducible neuroimaging stage-specific fingerprints.

In relation to the ML application, the endpoints include: the development and optimization of a supervised ML algorithm to predict clinical stage transitions based on clinical, biological, and neuroimaging data; the evaluation of ML predictive performance through cross-validation techniques (e.g., leave-one-out validation); and the external validation of ML-based predictions against independent clinical stage assignments made by expert psychiatrists.

## 4 Methods and data collection

### 4.1 Study participants

The broad inclusion and exclusion criteria (**Table 1**) include subjects with a variety of manifestations of the bipolar spectrum, from having a first-degree relative with BD to a clinical diagnosis of full-blown BD. Both genders aged between 18 and 70 years have been included. Furthermore, MRI-specific exclusion criteria were established (**Table 2**).

**Table 1:** Inclusion/exclusion criteria for BOARDING-PASS study entry.

Inclusion criteria
1. A range of bipolar spectrum manifestations meeting the criteria of the Kupka & Hillegers’ staging model
2. Both genders
3. Age ≥18 and ≤70 years
4. Written informed consent obtained

Exclusion criteria
1. Inability to provide informed consent
2. Diagnosis of intellectual disability
3. Presence of a severe medical condition (e.g., previously diagnosed neurological disorders, including chronic migraine, hematological diseases, renal disorders, history of stroke, or diabetes mellitus, even if compensated; controlled hypertension allowed)
4. Current substance use disorder or within six months prior to screening

**Table 2:** MRI-specific exclusion criteria.

Exclusion criteria
1. Claustrophobia
2. Presence of metallic prostheses or retained metal fragments in the body
3. Presence of cardiac or neurological stimulators, surgical clips, or cochlear/ocular implants
4. Female participants who are pregnant or in the postpartum period
5. History of traumatic brain injury with loss of consciousness

Participants provided their written informed consent to enter in the study and for the use of their anonymized data for research purposes. The study protocol will be conducted in accordance with the ethical standards of the relevant national and institutional committees on human experimentation and with the Helsinki Declaration of 1975, as revised in 2008 (PMC2566407). The Ethics Committee of Milan, Area 1, reviewed and approved the study (N.0008265/2023 on 24/02/2023).

### 4.2 Stage assignment

At baseline and at all subsequent time points, stages will be assigned according to the Kupka and Hillegers’ model, reported in **Table 3** and herein summarized: stage 0 (increased risk, i.e., having a first-degree relative with BD, without psychiatric symptoms); stage 1 (presence of nonspecific psychiatric symptoms or depressive episode[s]); stage 2 (first mood episode meeting criteria for BD diagnosis); stage 3 (recurrent depressive, hypomanic, manic, or mixed episodes); and stage 4 (persistent, unremitting illness with chronic depressive, manic, or mixed episodes, including rapid cycling). In particular, for subjects classified as stage 1 according to the Kupka & Hillegers’ model, we referred to the specific operational criteria defined by Bechdolf et al. (45), described in **Table 4**.

**Table 3:** Staging model by Kupka & Hillegers.

STAGE 0	↑ risk (as defined by a 1^st^ degree relative with BD^‡^); no psychiatric symptoms
STAGE 1	Non-specific psychiatric symptoms or depressive episode(s)
1A	↑ risk and non-specific psychiatric symptoms, no history of depressive episode(s)
1B	↑ risk and bipolar-specific prodromal symptoms, no history of depressive episode(s)
1C	↑ risk, with a first MDE^†^
1D	↑ risk, with recurrent MDEs^†^
STAGE 2	1^st^ episode that qualifies for diagnosis of BD
2A	1^st^ manic episode (BD^‡^ I diagnosis) without previous history of depressive episode(s) and without depression immediately preceding or following 1^st^ manic episode
2B	1^st^ hypomanic (BD^‡^ II diagnosis) or manic episode (dx BD^‡^ I) without previous history of depressive episode(s) but with depression immediately preceding or following 1^st^ (hypo)manic episode
2C	1^st^ hypomanic (BD^‡^ I diagnosis) or manic episode (dx BD^‡^ I) with previous history of depressive episode(s), with or without depression immediately preceding or following 1^st^ (hypo)manic
2D	1^st^ depression after hypomanic episode (BD^‡^ II diagnosis)
STAGE 3	Recurrence of any depressive, hypomanic, or manic/mixed episode
3A	Recurrence of subsyndromal depressive or manic symptoms after the diagnosis of BD^‡^
3B	Recurrent BD^‡^ (recurrence of any depressive, hypomanic, or manic/mixed episode) and with full symptomatic and functional recovery between episodes
3C	Recurrent BD^‡^ (recurrence of any depressive, hypomanic, or manic/mixed episode), with subsyndromal symptoms and/or impaired functioning between episodes
STAGE 4	Persistent unremitting illness; chronic (> 2 years) depressive, manic or mixed episodes, including rapid cycling
4A	Chronic depressive, manic or mixed episode(s), without symptomatic and functional recovery for 2 years
4B	Rapid cycling (≥ 4 mood episodes/year), without symptomatic and functional recovery for 2 years

↑: increased; †MDE: Major Depressive Episode; ‡BD: Bipolar Disorder.

**Table 4:** At-risk criteria for bipolard disorder (adapted from 45).

	Clinical Manifestations
Group I: Subthreshold Mania	At least two consecutive days (but <4 days): abnormally elevated/irritable mood plus ≥ 2 of the following: decreased need for sleep, pressured speech, flight of ideas, distractibility, increased goal-directed activity, psychomotor agitation.
Group II: Depression + Cyclothymic features	Depressive episode ≥ 1 week (depressed mood or anhedonia + ≥ 2 depressive symptoms); plus recurrent brief episodes with subthreshold manic symptoms (lasting <4 days but ≥2 days, or very brief episodes recurring ≥4 days lifetime).
Group III: Depression + Genetic Risk	Depressive episode ≥1 week (depressed mood or anhedonia + ≥2 depressive symptoms); plus presence of bipolar disorder diagnosis in a first-degree relative.

### 4.3 Screening and clinical data

Structured forms will be used for systematic sociodemographic and clinical data collection, including:

- sociodemographic characteristics: age, gender, ethnicity, education level, marital status, and employment status.
- clinical variables: family psychiatric history, BD subtype, age at BD onset, stressful life events at onset, duration of illness, duration of untreated illness, age at first depressive/manic/hypomanic episode, polarity of first and most recent episode, predominant polarity, number of lifetime episodes, mixed or rapid cycling presentations, current and previous treatments, medical and psychiatric comorbidities, history of substance use disorder, number of lifetime hospitalizations and suicide attempts. The above-mentioned variables will be assessed at baseline (T0) and some of them (i.e., number of lifetime episodes, presence of mixed or rapid cycling features, current treatments, number of lifetime hospitalizations and suicide attempts) will be evaluated at each subsequent time point to track potential stage progression. It is worth noting that participants receiving pharmacological treatment, as well as those initiating treatment during the study, are eligible for inclusion only if they have maintained at least four weeks of pharmacological stability (i.e., the absence of significant changes in therapeutic class, dosage, or regimen) at both study entry and each follow-up assessment. This criterion will be applied to all participants, regardless of treatment status or clinical stage. In addition, both current, past, and newly initiated pharmacological treatments will be systematically recorded in detail, including therapeutic class, dosage, treatment duration, and adherence. Any changes in therapeutic strategies, such as initiation, discontinuation, dosage adjustments, switching of medications, or introduction of adjunctive psychotherapeutic interventions, will also be documented at each time point.
Moreover, structured clinical interviews and a battery of psychometric measures and questionnaries will be administered, as follows (see **Table 5**):
- *Structured Clinical Interview for DSM-5, Research Version (*SCID-5-RV; 46 *):* a standardized, semi-structured interview specifically developed for systematic diagnostic assessment according to DSM-5 criteria (47) in research settings. This version allows for rapid and reliable diagnostic assessment of major psychiatric disorders, typically requiring approximately 30 minutes to complete. It will be administered during the screening phase by trained clinicians.
- *Family Interview for Genetic Studies* (FIGS, 48): a structured clinician-administered interview, administered at the screening phase, to collect comprehensive information on psychiatric family history, including the presence of BD among first-degree relatives. Time of administration has been estimated in approximately 15 minutes per family member.
- *Drugs Abuse Screening Test* (DAST-10, 49): a 10-item, self-report questionnaire, filled out at the screening, to detect the presence and the severity of substance abuse. Administration time has been estimated in nearly 5 minutes.
- *TWEAK test* (50): a 5-item self-report screening scale for alcohol abuse, performed at the screening phase, with a completion time of roughly 2 minutes.
- *Test Intelligenza Breve* (TIB, 43): the Italian adaptation of the National Adult Reading Test (NART; 52), will be administered at baseline in approximately 10 minutes, provides an estimate of premorbid intelligence quotient (IQ) comparable to scores obtained with other intelligence tests (e.g., the Wechsler Adult Intelligence Scale-WAIS; 53).
- *Hamilton Depression Rating Scale* (HDRS 21, 54): a 21-item clinician-administered scale which measures the severity of depression, with a focus on somatic symptoms. Its time of administration has been estimated in approximately 20 minutes and will be administered at each time point.
- *Montgomery Asberg Depression Rating Scale* (MADRS, 55): a 10-item clinician-administered scale assessing depressive symptoms, performed at each time point in nearly 15 minutes.
- *Hamilton Anxiety Rating Scale* (HAM-A, 56): a 14-item clinician-administered scale evaluating the severity of anxiety symptoms, including both psychological and somatic domains. Its administration, at every time point, has been estimated in around 15 minutes.
- *Young Mania Rating Scale* (YMRS, 57): an 11-item clinician-administered scale assessing the severity of manic symptoms, completed in about 15 minutes and administered at each time point.
- *Sheehan Disability Scale* (SDS, 58): a 5-item self-report scale measuring functional impairment in work, social and family domains, will be administered in around 5 minutes at each time point.
- *Global Assessment of Functioning* (GAF, 59): a clinician-rated scale providing an overall measure of psychiatric illness severity and functional impairment, completed in approximately 2 minutes and rated at each time point.
- *Childhood Trauma Questionnaire* (CTQ, 60): a 28-item self-report instrument designed to assess experiences of abuse and/or neglect during childhood. It evaluates five distinct domains: emotional abuse, physical abuse, sexual abuse, emotional neglect, and physical neglect. It requires 5–10 minutes and will be administered at baseline.
- *Paykel Scale for Recent Life Events* (61): a semi-structured, clinician-administered interview assessing the occurrence and subjective impact of 61 major stressful life events across interpersonal, occupational, financial, and health-related domains over the prior six months. It is widely employed in psychiatric research to investigate the role of recent life stressors in the onset or exacerbation of psychiatric disorders. Its administration takes approximately 15 minutes and will be conducted at baseline.
- *Clinician Rating Scale* (CRS, 62): a clinician-administered scale, recorded in nearly 2 minutes at each time point and aimed at evaluating patients’ adherence to pharmacological treatment.

**Table 5:** Clinical assessment battery and administration schedule.

Domain	Measure	Method	Assessment timepoints
Diagnostic screening	Structured Clinical Interview for DSM-5, *Research version* (SCID-5-RV)	Clinician-rated	Screening phase
Family history	Family Interview for Genetic Studies (FIGS)	Clinician-rated	Screening phase
Substance use	Drug Abuse Screening Test (DAST-10)	Self-reported	Screening phase
	TWEAK Test (alcohol abuse screening)	Self-reported	Screening phase
Cognitive	Test Intelligenza Breve (TIB, premorbid IQ estimation)	Clinician-rated	T0
Symptoms	Hamilton Depression Rating Scale (HDRS-21)	Clinician-rated	T0, T1, T2 and T3
	Montgomery-Asberg Depression Rating Scale (MADRS)	Clinician-rated	T0, T1, T2 and T3
	Young Mania Rating Scale (YMRS)	Clinician-rated	T0, T1, T2 and T3
	Hamilton Anxiety Rating Scale (HAM-A)	Clinician-rated	T0, T1, T2 and T3
Functioning	Sheehan Disability Scale (SDS)	Self-reported	T0, T1, T2 and T3
	Global Assessment of Functioning (GAF)	Clinician-rated	T0, T1, T2 and T3
Childhood trauma	Childhood Trauma Questionnaire (CTQ)	Self-reported	T0
Recent stressful events	Paykel Scale for Recent Life Events	Clinician-rated	T0
Treatment adherence	Clinician Rating Scale (CRS)	Clinician-rated	T0, T1, T2 and T3

T0 = baseline; T1 = 6 months; T2 = 12 months; T3 = 18 months.

### 4.4 Gene transcription regulation, inflammation, microbiome data

Biological samples for gene expression, inflammation, and microbiome analyses will be collected at baseline, T2 and T3. Specifically:

- unstimulated saliva samples —i.e., whole saliva collected under resting conditions without gustatory, masticatory, or pharmacological stimulation— will be obtained using cotton buccal swabs (Salivette, Sarstedt, Nümbrecht, Germany) and stored at -20 °C until genomic DNA (gDNA) extraction. Exosomes wil be also isolated from saliva and miRNAs purified using an exosome RNA isolation kit.
- peripheral venous blood samples will be collected in two 5 ml vacutainer tubes containing sodium citrate. Serum and cellular components will be separated and total RNA as well as gDNA will be extracted from PBMCs.

All biological samples collected at the three recruiting sites (Units 1, 2 and 3) will be transferred to the central laboratory (Unit 4) for standardized processing and analyses, including:

- LIPIDOMICS to analyze short chain fatty acids (SCFAs) extracted from saliva derivatization for LC–MS/MS analysis will be carried out (42).

Molecular biology studies:

- gene expression analysis. Relative abundance of mRNA species in PBMCs will be assessed by real-time RT-PCR and Digital PCR (63).
- DNA methylation in both blood and saliva cells a. general DNA methylation status will be analyzed using the Reduced representation bisulfite sequencing (RRBS) method (SBS sequencing of the SURFseq5000 platform); b. gene-specific DNA methylation study will be performed on amplified bisulfite (BS) treated DNA and methylation levels analyzed using PyroMark Q48 (64).
- salivary exosomal miRNAs: miRNOme analysis (65) and selected miRNAs after networking analysis by RealTime PCR and Digital PCR (66).
- Transcriptional factors DNA-binding. ALPHAScreenTM assay technique to verify if identified recognition elements at genes promoter bind to different transcriptional factors and if this binding is directly modulated by the methylation degree of CpG motifs (67).
- Salivary MICROBIOTA COMPOSITION by 16S rRNA Microbiome sequencing (65).

### 4.5 Neuroimaging data

MRI assessments will be performed using 3T scanners at T0, T2, and T3, and comprised:

**-** Structural MRI (sMRI): 3D T1-weighted images will be acquired using a SPGR sequence (TE = minimum (full); flip angle, 6°; FOV, 250 mm; bandwidth, 31.25; matrix, 256 x 256) with 124 axial slices of 1.3 mm thickness. Following cortical surface reconstruction, local gyrification indices will be computed for 68 parcellated cortical regions based on the Desikan Atlas using FreeSurfer v7.1.0. A jackknife bias estimation procedure will then be applied to determine each individual’s contribution to group-level covariance structure, generating a 68×68 individual-wise distance matrix. The topological organization of the resulting structural covariance networks will subsequently be analyzed using the Graph Analysis Toolbox (68).

- Resting-state functional MRI: rs-fMRI images will be acquired using a gradient-echo EPI sequence with 36 axial slices (TE = 30 ms; TR = 2000 ms; voxel size: 3×3×4 mm^3^; matrix size: 64× 64; FOV: 192×192 mm^2^);, acquired in interleaved order. Each resting-state session will consist of 400 volumes. Pre-processing will be conducted using a combination of FMRIB’s Software Library (FSL) and custom MATLAB scripts. The pipeline will include the following steps: (1) reorientation to standard space; (2) detection of outlier volumes, followed by spline-based interpolation of outlier timepoints; (3) spatial and temporal preprocessing, including motion correction (MCFLIRT), temporal high-pass filtering, and spatial smoothing (FWHM = 5 mm); (4) brain extraction of the structural image; (5) nonlinear registration to the MNI152 standard space using FSL-FNIRT. Static and dynamic functional connectomes will be estimated by calculating z-transformed Pearson correlation coefficients between all pairs of brain regions in the adopted parcellation scheme. Dynamic connectivity will be computed using a sliding-window approach with a window length of 30 TRs and a step size of 2 TRs. These steps will be implemented through in-house software developed in MATLAB. Graph-theoretical measures will be computed through the Brain Connectivity Toolbox (MATLAB, 69).

**Figure 1:**
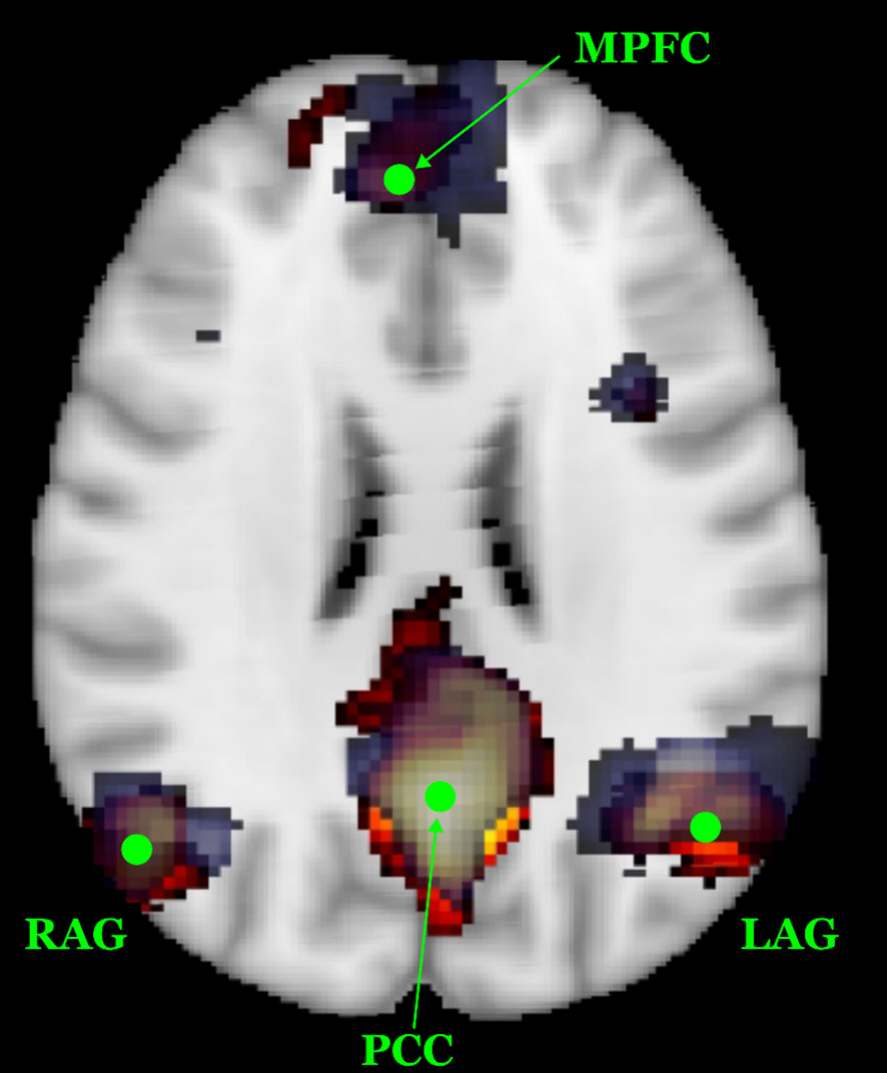
Spatial reproducibility of the DMN topography in the travelling subject across the two MRI sites. The DMN component derived from UO3 is shown on a blue scale, while the component from UO2 is displayed in a red-yellow scale. Green dots indicate the primary DMN regions of interest, i.e. posterior cingulate cortex - PCC, medial prefrontal cortex - MPFC, left and right angular gyri – L/RAG. The substantial spatial overlap highlights good inter-site consistency.

To minimize inter-site variability in neuroimaging data, both structural and functional MRI acquisitions were performed using harmonized protocols across the two imaging centers, each equipped with a 3 T scanner. Additionally, we employed a travelling subject scanned at both sites to directly assess cross-site consistency. The resulting structural and functional datasets from this subject demonstrated good reproducibility. In particular, the spatial topography of the Default Mode Network (DMN) was used as a preliminary benchmark for inter-site comparability. As shown in **Figure 1**, the DMN topography derived from each site (UO3 in blue; UO2 in orange) largely overlap, with consistent identification of the main DMN regions (posterior cingulate cortex, medial prefrontal cortex, left and right angular gyri, shown as green markers) While these preliminary results are encouraging, *post hoc* harmonization techniques such as ComBat may be considered during further analysis (39, 71, 72).

### 4.6 ML algorithms

A ML framework will be developed to predict clinical stage transitions in BD by integrating collected clinical, biological, and neuroimaging data. To manage the integration of multimodal data, we will adopt robust pre-processing pipelines including data normalization, outlier detection, and imputation methods for handling missing values (e.g., k-nearest neighbor or multiple imputation, 73). ML analyses will start at month 6 of the study and will be conducted using MATLAB’s Statistics and Machine Learning Toolbox, initially supported by NeuroMiner software (http://proniapredictors.eu/neurominer/index.html; 74), a validated software platform designed to manage heterogeneous datasets. NeuroMiner provides a broad range of cross-validation frameworks, preprocessing strategies, supervised learning algorithms, feature selection tools, and external validation methods. The ML approach will be based on supervised classifiers, primarily Support Vector Machine (SVM) and Bayesian models. In the preliminary phase, classifiers will be trained and tested on preliminary datasets to compare alternative predictive models in a controlled setting and to identify the best-performing algorithmic configuration. Subsequently, feature selection procedures will be employed to identify the most discriminative variables from the pool of candidate features. This step is crucial to enhance model interpretability and to prevent overfitting, especially in small-sample, high-dimensional datasets typical of multimodal studies. In fact, by reducing the number of input variables, feature selection minimizes noise, lowers model complexity, and improves generalizability of the ML predictions. If needed (i.e. if the dimensionality of the feature space is still too high), to further reduce overfitting and the computational burden, Principal Component Analysis might be applied. SVM classifiers will be prioritized due to their robustness in handling high-dimensional, small-sample data. In addition, class weighting will be applied so that errors on minority stages are penalized more heavily, reducing the bias introduced by uneven group sizes. Model performance will be assessed using cross-validation techniques (e.g. leave-one-out or stratified k-fold, depending on data structure and class distribution) and evaluated in terms of sensitivity, specificity, F1 score and overall accuracy, in order to account for potential class imbalance. A predefined minimum target accuracy of 90% is required for model deployment on the full dataset. For external validation, ML-derived stage predictions will be compared against those assigned by expert clinicians, considered the diagnostic gold standard. An interim evaluation of model accuracy has been planned after acquisition of 50% of the total dataset, serving as a critical checkpoint to assess the predictive performance of the model and to estimate its expected accuracy upon completion of data collection. This intermediate analysis is particularly relevant should the timeframe between data acquisition completion and the project conclusion proves insufficient for final model training and validation.

## 5 Statistical analysis and management

For stage progression analysis, data from repeated assessments of clinical stages across follow-up visits will be analyzed using generalized estimating equations implemented via the PROC GENMOD procedure in SAS^®^, applying a multinomial distribution with a logit link function. This approach allows evaluating associations and interactions between stage transitions and relevant sociodemographic and clinical variables over time. Additionally, stage transition probabilities will be modeled using a multi-state Markov approach provided by the ‘mstate’ package in R. Statistical significance was set at p≤.05. Biological and neuroimaging data analyses will be performed by linear mixed-effects models to evaluate the associations between biomarkers and neuroimaging outcomes across BD stages at different time points. Omnibus one-way ANOVA will be used for molecular measures, complemented by *post-hoc* analyses for multiple comparisons when significant effects are identified. ML analyses will be conducted using MATLAB’s Statistics and Machine Learning Toolbox, supported by NeuroMiner software (74).

## 6 Sample size, power, and effect size

Sample size estimation was guided by multiple methodological considerations. Given the multidimensional nature of the study, several independent measures - clinical, biological, and neuroimaging - will be collected longitudinally and subsequently integrated into a unified ML predictive model. Therefore, the sample size was determined to ensure sufficient statistical power for traditional analyses, while also supporting the training and validation of robust ML algorithms. Specifically, the required sample size has been determined based on two complementary criteria. First, among the multiple domains investigated, a power analysis conducted using G*Power 3.1.9.7 (75) indicated that a total of N = 126 participants was sufficient to detect significant differences across five BD stages (0, 1, 2, 3, 4), assuming a statistical power of 0.80, an alpha=0.05, and a medium effect size (f=0.32). Second, from a computational perspective, this sample size was considered adequate for training supervised ML models with acceptable performance. Previous ML-based studies in BD have shown that models trained on ~50 subjects reached an accuracy of ~64%, while those trained on samples approaching 90 participants achieved accuracy up to 99% (76). Based on these observations and accounting for an estimated dropout rate of 15%, a total sample size of 126 participants was deemed both feasible and methodologically robust, ensuring sufficient accuracy of the ML models.

## 7 Study organization

### 7.1 Study sites and organizational structure

BOARDING-PASS study will be conducted across four operational units (UOs), each with defined roles to ensure high-quality data acquisition and standardized clinical procedures.

- UO1 (ASST Fatebenefratelli-Sacco, Milan) is the coordinating center, responsible for patient recruitment and baseline diagnostic assessments, standardized collection of clinical and biological data. UO1 investigators will provide coordination and oversight within the multicenter research network, maintaining effective communication among clinicians, researchers, and technical staff.
- UO2 (ASST Papa Giovanni XXIII, Bergamo) is responsible for participant recruitment, diagnostic assessment, collection of biological samples, structural and resting-state MRI data acquisition also on behalf of UO1, and structural neuroimaging analysis.
- UO3 (ASL 2 Lanciano-Vasto-Chieti) will conduct participant recruitment and assessment, data collection and management, biological sample collection, as well as structural and functional neuroimaging data acquisition.
- UO4 (University of Teramo) serves as the centralized facility responsible for biological analyses, specifically gene transcription regulation and microbiota analyses. UO4 is also responsible for the analysis of functional neuroimaging data and for the implementation of ML algorithms.

### 7.2 Site selection/training/recruitment

All research personnel across participating sites, including psychiatrists, psychologists, and researchers, underwent standardized training prior to the study initiation. This training ensured homogeneous adherence to protocol-specific procedures for diagnostic interviewing, psychometric assessments, biological sample collection, and neuroimaging acquisition and processing. To maintain methodological rigor and inter-site consistency, periodic retraining sessions and quality assurance audits will be implemented throughout the study duration.

## 8 Current status

As of May 2025, a total of 126 subjects had been enrolled in the study. Among them, 54 completed T1 and 25 completed T2. At present, T1 and T2 assessments are ongoing and T3 is scheduled to begin next July. The current dropout rate is below 10%, consistent with our initial expectations. Preliminary internal tests on partial datasets suggest promising accuracy levels (>70%) in stage prediction using supervised ML models. These results, although not formally included in the current manuscript, support the feasibility of our analytical pipeline and will be detailed in future dedicated publications.

## 9 Discussions and conclusions

Although several staging models for BD have been conceptualized to date, current classifications still rely predominantly on clinical symptomatology, with limited integration of biological and neuroimaging correlates of illness progression. Incorporating these dimensions may significantly enhance the patient characterization by identifying specific dysfunctional trajectories and overcoming the traditional, diagnosis-centered approaches, ultimately leading to more targeted and effective treatment strategies. Moreover, the application of ML techniques may implement the accuracy in stage classification, enabling the development of data-driven and personalized staging systems. BOARDING PASS study aligns with the growing need for a standardized and transdiagnostic staging framework that integrates immuno-inflammatory, epigenetic, and neuroimaging biotypes. Such an approach is crucial to enhance the validity of staging in BD and to optimize its long-term clinical management. Preliminary results, after having successfully completed the initial planned sample recruitment, are expected for late 2026. Future directions may consider longer time of assessment and the inclusion of a matched control group in order to strengthen the study design and scientific validity of the findings.
